# The Impact of Digital Health on Smoking Cessation

**DOI:** 10.2196/41182

**Published:** 2023-03-15

**Authors:** Raquel Cobos-Campos, Jose Aurelio Cordero-Guevara, Antxon Apiñaniz, Arantza Sáez de Lafuente, Cristina Bermúdez Ampudia, Julene Argaluza Escudero, Iraida Pérez Llanos, Naiara Parraza Diez

**Affiliations:** 1 Epidemiology and Public Health Group Bioaraba Health Research Institute Vitoria-Gasteiz Spain; 2 Osakidetza Basque Health Service Vitoria-Gasteiz Spain; 3 Department of Preventive Medicine and Public Health University of the Basque Country Vitoria-Gasteiz Spain; 4 Research Network on Chronicity, Primary Care and Health Promotion Madrid Spain

**Keywords:** smoking cessation, smoking, cessation, smoker, quit, care delivery, service delivery, health technology, mHealth, mobile applications, mobile health, digital health, mobile app, health app, smartphone, health service, eHealth, trend

## Abstract

**Background:**

Smartphones have become useful tools for medicine, with the use of specific apps making it possible to bring health care closer to inaccessible areas, continuously monitor a patient's pathology at any time and place, promote healthy habits, and ultimately improve patients’ quality of life and the efficiency of the health care system. Since 2020, the use of smartphones has reached unprecedented levels. There are more than 350,000 health apps, according to a 2021 IQVIA Institute report, that address, among other things, the management of patient appointments; communication among different services or professionals; the promotion of lifestyle changes related to adopting healthy habits; and the monitoring of different pathologies and chronic conditions, including smoking cessation. The number of mobile apps for quitting smoking is high. As early as 2017, a total of 177 unique smoking cessation–relevant apps were identified in the iPhone App Store, 139 were identified in Google Play, 70 were identified in the BlackBerry app store, and 55 were identified in the Windows Phone Store, but very few have adequate scientific support. It seems clear that efforts are needed to assess the quality of these apps, as well as their effectiveness in different population groups, to have tools that offer added value to standard practices.

**Objective:**

This viewpoint aims to highlight the benefits of mobile health (mHealth) and its potential as an adjuvant tool in health care.

**Methods:**

A review of literature and other data sources was performed in order to show the current status of mobile apps that can offer support for smoking cessation. For this purpose, the PubMed, Embase, and Cochrane databases were explored between May and November 2022.

**Results:**

In terms of smoking cessation, mHealth has become a powerful coadjuvant tool that allows health workers to perform exhaustive follow-ups for the process of quitting tobacco and provide support anytime and anywhere. mHealth tools are effective for different groups of smokers (eg, pregnant women, patients with chronic obstructive pulmonary disease, patients with mental illness, and the general population) and are cost-effective, generating savings for the health system. However, there are some patient characteristics that can predict the success of using mobile apps in the smoking cessation process, such as the lower age of patients, dependence on tobacco, the number of quit attempts, and the previous use of mobile apps, among others. Therefore, it is preferable to offer these tools to patients with a higher probability of quitting tobacco.

**Conclusions:**

mHealth is a promising tool for helping smokers in the smoking cessation process. There is a need for well-designed clinical studies and economic evaluations to jointly assess the effectiveness of new interventions in different population groups, as well as their impact on health care resources.

## Introduction

The health service sector is one of the most complex existing sectors [1], and although we are witnessing how new technologies are changing industries, business models, and markets in a disruptive way within just a few years or months, this complexity explains why the health sector is slower when it comes to adapting to this evolving environment [2]. The data we handle in health care are sensitive data that are subject to exhaustive data protection regulation, which makes accessing and expanding health care technology difficult. Nevertheless, strategies for the use of information and communication technologies in the health sector have been gaining ground, and there is now a majority consensus on the fundamental role of these technologies in improving the efficiency and accessibility of the health system [2]. Technological advances are changing all aspects of society, improving and speeding up processes with the aim of improving people's quality of life [2].

The health care sector is no stranger to these changes, and many technological innovations are making increasingly useful services and tools available [3]. Among these innovations, big data and artificial intelligence (AI) have become promising tools for the management of chronic diseases [4] and enable the use of innovative and promising diagnostic and therapeutic applications [5]. AI is understood as a working methodology for compiling an enormous amount of information (known as *big data*) in order to subsequently use powerful computer programs to try to obtain data on decision-making elements in all areas of life [5].

Web 3.0 is the next generation of internet technology that relies on the use of AI to process data and create a personalized user experience [6]. Given the large amount of information and metadata that are being generated and made publicly available, it is believed that Web 3.0 technologies (eg, machine learning, AI, Internet of Things, and natural language processing) will allow computer agents to automatically link any kind of data from any system to build inferences from those data [6].

In addition to the above, another current trend is the use of mobile health (mHealth), which is defined by the World Health Organization as the use of mobile devices, such as smartphones and patient monitoring devices, for medical practice and public health [7].

Smartphones have become useful tools for medicine, with the use of specific apps making it possible to bring health care closer to inaccessible areas, continuously monitor a patient's pathology at any time and place, promote healthy habits, and ultimately improve the quality and efficiency of the health care system [3].

This improvement of health care process quality is the result of several factors, such as the remote monitoring of patients, which makes it possible to predict potential problems early and take the necessary measures within a sufficient time frame, thereby reducing the number of unnecessary consultations and hospitalizations. This allows practitioners to focus on investing their time in solving important health problems that cannot be solved remotely [3].

One of the United Nations Sustainable Development Goals for 2030 is “to ensure healthy lives and promote well-being for all at all ages” [8]. However, the World Health Organization states that universal health coverage will not be achieved without the support of eHealth [9].

There are already more mobile devices than people in the world today [10]. According to the Global System Mobile Association, there are more than 9.5 billion mobile connections [11], while the worldwide population consists of more than 7.9 billion people [12].

Since 2020, the use of smartphones has reached unprecedented levels. In 2021, this use grew by 30% when compared to that in 2020. Moreover, according to App Annie's *State of Mobile 2022* report [13], 230,000 new apps were downloaded in 2021—a 5% increase from 2020. The amount of health-related mobile apps is starting to reach considerable numbers. There are currently more than 350,000 health apps, according to a 2021 report by the IQVIA Institute [14], aimed at, among other things, the promotion of lifestyle changes related to adopting healthy habits, the monitoring of different pathologies, the management of patient appointments, and communication among different services or professionals [15]. It is estimated that around 30% of health apps are targeted toward health care professionals and 70% are targeted toward the general population [15]. Self-care through mobile devices is another growing field [16].

Of these apps, very few have the necessary quality that should be demanded from such tools, which has resulted in greater noise and difficulty in selecting apps that can add value to people's lives [15,17-19]. The Andalusian Health Quality Agency developed extensive guidance on the criteria [20] that a good health app should meet. Some of these criteria are as follows: *relevance* (it is clearly defined what the app is for, what its objectives are, and who it is aimed at), *testing* (the app has been tested beforehand on the target audience), *transparency* (authors, funding sources, and conflicts of interest are clearly identified), *content and sources* (the health app is based on reliable sources and available scientific evidence and specifies when the information was last updated), and *risk management* (risks that may be associated with the use of the app are identified). It is necessary to involve end users (both health care professionals and patients) in app design to ensure greater quality and usability [20] and, once the app is designed, evaluate app efficacy. There is now ample evidence about the utility of mHealth in different contexts, such as increasing the rate of consultation attendance [21], promoting safer sex [22], monitoring patients with diabetes, managing low back pain [23,24], and treating smoking dependence. The National Institute for Health and Care Excellence (NICE) considers digital and mHealth interventions as options for helping people stop smoking and adjuncts to existing services [25]. The NICE also advises that text message–based interventions that use tailored messages may be more effective than other digital health and mHealth interventions [25].

Smoking is one of the main causes of global morbidity and mortality and a risk factor of a high number of chronic diseases, such as cancer, cardiovascular disease, and chronic obstructive pulmonary disease (COPD), among others. The life span of smokers is, on average, about 10 years shorter than that of nonsmokers. However, quitting smoking can increase life expectancy, and the number of years of life gained depends on the age at which a person quits smoking [26]. Tobacco kills more than 8 million people per year, of whom more than 7 million are direct users and about 1.2 million are nonsmokers exposed to secondhand smoke [27]. Furthermore, the adverse health consequences of tobacco are well known and have major economic implications [28]. The World Bank estimated that high-income countries spend 6% to 15% of their total health expenditure on tobacco-related diseases [29]. Smokers have higher rates of absenteeism and longer absences from work than those of nonsmokers due to the higher prevalence of tobacco-related diseases among smokers [28].

There are currently different approaches to smoking cessation treatment, such as more intensive or less intensive motivational counseling–based interventions and pharmacological therapy [30]. The mobile telephony boom and, in recent years, the increase in the number of mobile apps, with a penetration of 100% in the world population, have provided new tools for helping both professionals and patients in the management of different pathologies, including smoking cessation.

According to the Survey on Alcohol and Other Drugs in Spain (Encuesta Sobre Alcohol y Otras Drogas en España 2019-2020), the average age of smoking initiation is 16.6 years [31]. With 50% and 29% of the population accessing their first smartphone at 11 to 12 years of age and between 13 and 14 years of age, respectively, mHealth is a great alternative tool for preventing, reducing, or quitting smoking in these age groups [32].

There are a large number of smokers who prefer to not use drugs to quit smoking. It is therefore clear that it is necessary to carry out interventions that do not imply the need for pharmacological treatment and to have data on efficacy and efficiency that support their generalization to the smoking population. Other reviews that address this topic have been performed [33-35]. However, this viewpoint paper is not so much a review in itself, but rather an update on the state of digital health in general and its impact on smoking cessation programs. The aim of this viewpoint paper is to provide readers with an overview of the usefulness of digital health and, in particular, mHealth as adjuvant tools in smoking cessation programs.

## Evidence of mHealth Focused on Smoking Cessation

There is enough scientific evidence about the great potential of using mHealth as a complement of usual treatment in smoking cessation.

Whittaker et al [33] reported a pooled relative risk of smoking cessation of 1.69 in a systematic review of 12 clinical trials that evaluated the efficacy of mobile phone–based interventions. Chen et al [34] concluded that interventions based on the internet, software, mobile phones, or other electronic tools increase the likelihood of quitting tobacco when compared to no intervention or the use of generic self-help material. Dahne et al [36] assessed asynchronous smoking cessation e-visits that were performed proactively through the electronic health records of adult smokers who were treated within primary care. After 3 months, e-visit participants, when compared with usual treatment participants, were 4.13 (95% CI 1.06-16.10; *P*=.04) times more likely to have reduced their number of cigarettes smoked per day by at least 50%. In a recent systematic review that evaluated the efficacy of digital interventions in randomized clinical trial studies of smoking cessation, 19 trials (15,472 participants) were included in the analysis, and the overall abstinence rate (percentage of participants who did not smoke during a follow-up period of at least 3 months) at the end point was 17.8% (95% CI 17%-18.7%); the authors concluded that digital health had a clear positive effect when compared to self-help guidelines or no intervention [35].

By analyzing results among different groups of patients, overall, studies have demonstrated that smoking cessation apps are feasible for use among people diagnosed with mental illness, especially those with a high score on the System Usability Scale [37]—a reliable tool for measuring the ease of use of a wide variety of products and services, including hardware, software, mobile devices, websites, and applications. However, it appears clearly that apps designed specifically for patients with schizophrenia or other mental illnesses may be more accessible and user-friendly [38], emphasizing the importance of end user involvement in app development.

In another systematic review that assessed the efficacy of mobile phone–based behavioral interventions in pregnancy to promote maternal and fetal health in high-income countries, the authors concluded that the utilization of mobile phone–based health behavior interventions in pregnancy demonstrates some correlation with positive beliefs, behaviors, and health outcomes [39].

These types of tools have also been proven to be effective in managing COPD. In a review published recently, the authors concluded that pharmacotherapy combined with behavioral interventions that are delivered via mHealth may be an effective, safe, accessible, and cost-effective strategy for helping smokers with COPD quit smoking [40].

Smoking cessation has become a ubiquitous intervention approach for which user engagement can be readily measured. Nearly 500 English-language smartphone apps for smoking cessation have been downloaded more than 33 million times since 2012 (R Nelson, Sensor Tower Inc, email, April 15, 2020). Higher user engagement in smartphone interventions for smoking cessation is predictive of cessation outcomes [41,42]. However, there are certain characteristics that either predict the time when an app will be used or predict that an app will not be used. The act of smoking up to one-half pack per day, the act of smoking the first cigarette within 5 minutes after waking, a higher mean acceptance of internal physical sensations, female sex, minority race (people of color), Hispanic ethnicity, and a history of smoking for 10 or more years are related to longer periods of app use [43].

Our research group obtained similar results in a randomized clinical trial conducted with 320 motivated smoking cessation patients and evaluated the effectiveness of a combined program (motivational counseling and reinforcement messages sent to mobile phones) versus motivational counseling alone (OR 2.329) at 12 months after baseline [44].

**Figure 1:**
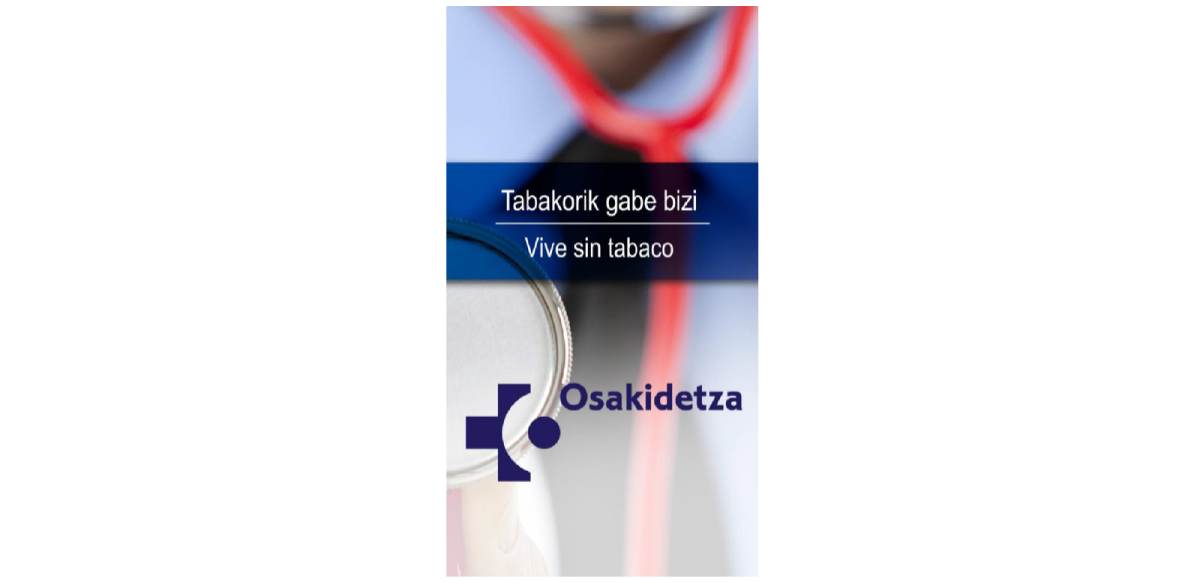
The *Vive sin tabaco* app.

These results allowed the transfer of the combined program to clinical practice after transforming the messaging program into a corporate app, which is available in all health centers of the Basque Public Health System (*Vive sin tabaco* app [Figure 1]). In parallel, a cost-effectiveness study was carried out to justify this transfer; the incremental cost-effectiveness ratio was calculated, with cost savings (from a societal perspective) of €5398 (US $5885.98) and €3290 (US $3587.42) per quality-adjusted life year gained for men and women, respectively [45]. In addition, a further cohort study was carried out with 92 patients who initiated a quit attempt with the *Vive sin tabaco* app, which showed smoking cessation results that were very similar to those of the previous clinical trial (14.1% vs 16.5% at 12 months) [46].

Quitting smoking can be a difficult challenge that sometimes requires many attempts before success is achieved. Nicotine dependence is a complex disorder [47]. However, the earlier smoking cessation occurs, the higher the number of life years regained [26]; therefore, it is of vital importance to encourage young people to not take up smoking and ensure that they internalize the benefits of not smoking. mHealth tools for smoking cessation have great potential for this age group.

Smoking cessation treatment is not only clinically effective but also cost-effective. Health advice is considered one of the most cost-effective interventions in the treatment of smoking [48]; however, the changes promoted by health advice do not last long [49]. Therefore, it is necessary to establish reinforcement mechanisms, among which are information and communication technologies and, more specifically, mHealth, for which there is ample evidence in the treatment of smoking.

## Discussion

### Principal Findings

Mobile technology has changed the way we live, work, and communicate. The use of mobile technologies to support the achievement of mHealth goals is an emerging and rapidly developing field that has the potential to play a key role in transforming health care to increase the quality and efficiency of care, and the mission of this field is to complement rather than replace traditional health care [50]. Health developments mainly include apps aimed directly or indirectly at maintaining or improving people's healthy behaviors, quality of life, and well-being [50].

Health care is transforming. Health care costs are rising, as health care must cope with the demand for increasingly personalized and long-term care. Moreover, it is estimated that the use of mobile apps could improve the efficiency of patient care and reduce the time spent accessing and analyzing information by up to 30% [51]. In fact, a study that was recently published in the *Journal of Medical Economics* concluded that patients who use digital health tools can reduce their monthly medical costs by around 22% [52].

Health apps are becoming technological tools with great potential for improving the way chronic diseases are managed. If they are well designed and focus on the needs of patients, they could more effectively facilitate the management of health care resources and communication between professionals and patients, thereby enhancing the active role of the population in their self-care [15].

Mobile apps have great potential to support patients in health care and encourage healthy behavioral changes. However, it is the features of apps that determine patients’ attitudes toward the use of apps, which in turn determine the success of apps [53]. Therefore, it is necessary that end users are involved at the beginning of the design process in order to increase the usability of apps.

Research has shown that demographics and personality characteristics are associated with the adoption and use of mobile apps. Income and level of education correlate positively with mobile phone use, whereas age correlates negatively with it [53]. The procurement of mHealth tools by older people is limited [54], and almost half (43%) of those aged over 70 years stop using them within the first 14 days [55] mainly due to the complexity of the tools [55], the limited health knowledge of users (ie, the knowledge required to fully understand the data) [55], and the cost of the technology [55]. This further emphasizes the need to design simple, end user–oriented tools and to involve end users in their design in order to obtain tools that fully meet end users’ expectations.

The number of quit attempts, nicotine dependence, the previous use of digital aids to quit smoking, and the Fagerstrom test score correlate with smokers’ attitudes toward the use of a smoking cessation app. However, different studies have found no significant relationship between demographic characteristics and attitudes toward or intentions to use a smoking cessation app [56,57]. Further, 77.5% of smokers who have used a mobile app to quit smoking have never checked the credibility of the developer or publisher of the health app [57].

It is clear that it is necessary to invest money and effort into having useful tools in health care that meet the expectations of end users and complement health care to increase the quality and efficiency of care. Mobile telephony was born to bring people together, but its purpose is now much greater than that.

### Conclusions

It seems clear that mHealth is a valuable tool that can provide support to both health professionals and patients in the complex process of smoking cessation. However, it is likely that several mHealth user characteristics predict the likelihood of the success of smoking cessation apps, such as age, tobacco dependence, and the number of cigarettes smoked per day, among others. Therefore, it would make sense to offer these apps to, for example, younger smokers, those who are more dependent on tobacco, and those who smoke more than half a pack of cigarettes per day.

## Abbreviations

AI: artificial intelligence
COPD: chronic obstructive pulmonary disease
mHealth: mobile health
NICE: National Institute for Health and Care Excellence

## References

[1] Institute of Medicine and National Academy of Engineering (2011). 6 next steps: Aligning policies with leadership opportunities. Engineering a Learning Healthcare System: A Look at the Future: Workshop Summary.

[2] Federación de Empresas de Tecnologías Sanitarias Hacia la transformación digital del sector de la salud. Sociedad Española de Informática de la Salud.

[3] Ambit-BST (2021). mHealth: todo lo que debes saber sobre la salud móvil. Ambit-BST.

[4] Majnarić LT, Babič F, O'Sullivan S, Holzinger A (2021). AI and big data in healthcare: Towards a more comprehensive research framework for multimorbidity. J Clin Med.

[5] Kedra J, Gossec L (2020). Big data and artificial intelligence: Will they change our practice?. Joint Bone Spine.

[6] Curchoe CL, Malmsten J, Bormann C, Shafiee H, Farias AFS, Mendizabal G, Chavez-Badiola A, Sigaras A, Alshubbar H, Chambost J, Jacques C, Pena CA, Drakeley A, Freour T, Hajirasouliha I, Hickman CFL, Elemento O, Zaninovic N, Rosenwaks Z (2020). Predictive modeling in reproductive medicine: Where will the future of artificial intelligence research take us?. Fertil Steril.

[7] The Competitive Intelligence Unit mHealth: Garantizar una vida sana y bienestar para todos. Squarespace.

[8] Department of Economic and Social Affairs Goal 3. United Nations.

[9] Global Observatory for eHealth (2016). Global diffusion of eHealth: Making universal health coverage achievable. World Health Organization.

[10] Fernández R (2022). Número de líneas de telefonía móvil por cada 100 habitantes en España 2016-2022. Statista.

[11] GSMA About us. GSMA.

[12] Worldometer Población mundial. Worldometer.

[13] data.ai State of mobile 2022. data.ai.

[14] Olsen E (2021). Digital health apps balloon to more than 350,000 available on the market, according to IQVIA report. MobiHealthNews.

[15] Iborra CR (2019). Las aplicaciones móviles de salud como herramientas de apoyo a la autogestión de cuidados del paciente crónico. Universidad Autónoma de Madrid.

[16] Fundación Telefónica (2021). Sociedad Digital en España 2020-2021. Fundación Telefónica.

[17] Salud Conectada Introducción, normativa y certificación de las Apps de Salud. Salud Conectada.

[18] Grau I, Kostov B, Gallego JA, Iii FG, Fernández-Luque L, Sisó-Almirall A (2016). [Assessment method for mobile health applications in Spanish: The iSYScore index]. Semergen.

[19] PICKASO Team (2018). Informe: El Uso de las Apps en España y en el Mundo en 2018. PICKASO.

[20] Agencia de Calidad Sanitaria de Andalucía Recomendaciones. Estrategia de calidad y seguridad en aplicaciones móviles para la salud.

[21] Marcolino MS, Oliveira JAQ, D'Agostino M, Ribeiro AL, Alkmim MBM, Novillo-Ortiz D (2018). The impact of mHealth interventions: Systematic review of systematic reviews. JMIR Mhealth Uhealth.

[22] Gold J, Aitken CK, Dixon HG, Lim MSC, Gouillou M, Spelman T, Wakefield M, Hellard ME (2011). A randomised controlled trial using mobile advertising to promote safer sex and sun safety to young people. Health Educ Res.

[23] Larsen ME, Turner J, Farmer A, Neil A, Tarassenko L (2010). Telemedicine-supported insulin optimisation in primary care. J Telemed Telecare.

[24] Rintala A, Rantalainen R, Kaksonen A, Luomajoki H, Kauranen K (2022). mHealth apps for low back pain self-management: Scoping review. JMIR Mhealth Uhealth.

[25] National Institute for Health and Care Excellence Tobacco: preventing uptake, promoting quitting and treating dependence. National Institute for Health and Care Excellence.

[26] Jha P, Ramasundarahettige C, Landsman V, Rostron B, Thun M, Anderson RN, McAfee T, Peto R (2013). 21st-century hazards of smoking and benefits of cessation in the United States. N Engl J Med.

[27] World Health Organization (2022). Tabaco. World Health Organization.

[28] Suárez-Bonel MP, Villaverde-Royo MV, Nerín I, Sanz-Andrés C, Mezquida-Arno J, Córdoba-García R (2015). Health care costs and work absenteeism in smokers: study in an urban community. Arch Bronconeumol.

[29] The W (1999). Curbing the epidemic: governments and the economics of tobacco control. The World Bank. Tob Control.

[30] Banegas JR, Díez-Gañán L, Bañuelos-Marco B, González-Enríquez J, Villar-Álvarez F, Martín-Moreno JM, Córdoba-García R, Pérez-Trullén A, Jiménez-Ruiz C (2011). [Smoking-attributable deaths in Spain, 2006]. Med Clin (Barc).

[31] Owens T (2022). At what age did you get your first smartphone?. Statista.

[32] Ministerio de Sanidad La Encuesta sobre alcohol y otras drogas en España, EDADES. Plan Nacional sobre Drogas.

[33] Whittaker R, McRobbie H, Bullen C, Borland R, Rodgers A, Gu Y (2012). Mobile phone-based interventions for smoking cessation. Cochrane Database Syst Rev.

[34] Chen YF, Madan J, Welton N, Yahaya I, Aveyard P, Bauld L, Wang D, Fry-Smith A, Munafò MR (2012). Effectiveness and cost-effectiveness of computer and other electronic aids for smoking cessation: a systematic review and network meta-analysis. Health Technol Assess.

[35] Sha L, Yang X, Deng R, Wang W, Tao Y, Cao H, Ma Q, Wang H, Nie Y, Leng S, Lv Q, Li X, Wang H, Meng Y, Xu J, Greenshaw AJ, Li T, Guo WJ (2022). Automated digital interventions and smoking cessation: Systematic review and meta-analysis relating efficiency to a psychological theory of intervention perspective. J Med Internet Res.

[36] Dahne J, Player M, Carpenter MJ, Ford DW, Diaz VA (2021). Evaluation of a proactive smoking cessation electronic visit to extend the reach of evidence-based cessation treatment via primary care. Telemed J E Health.

[37] Usability.gov System Usability Scale (SUS). Usability.gov.

[38] Sawyer C, Hassan L, Guinart D, Agulleiro LM, Firth J (2022). Smoking cessation apps for people with schizophrenia: How feasible are m-Health approaches?. Behav Sci (Basel).

[39] Hussain T, Smith P, Yee LM (2020). Mobile phone-based behavioral interventions in pregnancy to promote maternal and fetal health in high-income countries: Systematic review. JMIR Mhealth Uhealth.

[40] Feng L, Lv X, Wang Y, Chu S, Dai Z, Jing H, Tong Z, Liao X, Liang L (2022). Developments in smoking cessation interventions for patients with chronic obstructive pulmonary disease in the past 5 years: a scoping review. Expert Rev Respir Med.

[41] Browne J, Halverson TF, Vilardaga R (2021). Engagement with a digital therapeutic for smoking cessation designed for persons with psychiatric illness fully mediates smoking outcomes in a pilot randomized controlled trial. Transl Behav Med.

[42] Bricker JB, Mull KE, Kientz JA, Vilardaga R, Mercer LD, Akioka KJ, Heffner JL (2014). Randomized, controlled pilot trial of a smartphone app for smoking cessation using acceptance and commitment therapy. Drug Alcohol Depend.

[43] Bricker JB, Mull KE, Santiago-Torres M, Miao Z, Perski O, Di C (2022). Smoking cessation smartphone app use over time: Predicting 12-month cessation outcomes in a 2-arm randomized trial. J Med Internet Res.

[44] Cobos-Campos R, de Larrinoa AAF, de Lafuente Moriñigo AS, Diez NP, Barandiaran FA (2017). Effectiveness of text messaging as an adjuvant to health advice in smoking cessation programs in primary care. A randomized clinical trial. Nicotine Tob Res.

[45] Cobos-Campos R, Mar J, Apiñaniz A, de Lafuente AS, Parraza N, Aizpuru F, Orive G (2021). Cost-effectiveness analysis of text messaging to support health advice for smoking cessation. Cost Eff Resour Alloc.

[46] Cobos-Campos R, Apiñaniz A, de Lafuente AS, Parraza N (2022). Development, validation and transfer to clinical practice of a mobile application for the treatment of smoking. Aten Primaria.

[47] Arias AC (2002). Dependencia de Nicotina: aproximacion a su manejo farmacologico. Rev Colomb Psiquiatr.

[48] López MJT, Pérez AMB, Barceló IB, Ortíz JMB, Galvis JG, Pérez MG, López RM, Sánchez J, Tomás E, Soria AV, Grupo de Atención al Tabaquismo (GAT) de SmuMFyC (2008). Actitud de los profesionales de Atención Primaria frente al tabaco. Semergen.

[49] Cummings SR, Rubin SM, Oster G (1989). The cost-effectiveness of counseling smokers to quit. JAMA.

[50] Gazdecki A 9 mobile technology trends For 2017 (infographic). Bizness Apps.

[51] Haskins BL, Lesperance D, Gibbons P, Boudreaux ED (2017). A systematic review of smartphone applications for smoking cessation. Transl Behav Med.

[52] Whaley CM, Bollyky JB, Lu W, Painter S, Schneider J, Zhao Z, He X, Johnson J, Meadows ES (2019). Reduced medical spending associated with increased use of a remote diabetes management program and lower mean blood glucose values. J Med Econ.

[53] Chevalking SKL, Allouch SB, Brusse-Keizer M, Postel MG, Pieterse ME (2018). Identification of users for a smoking cessation mobile app: Quantitative study. J Med Internet Res.

[54] Peek STM, Luijkx KG, Vrijhoef HJM, Nieboer ME, Aarts S, van der Voort CS, Rijnaard MD, Wouters EJM (2019). Understanding changes and stability in the long-term use of technologies by seniors who are aging in place: a dynamical framework. BMC Geriatr.

[55] Puri A, Kim B, Nguyen O, Stolee P, Tung J, Lee J (2017). User acceptance of wrist-worn activity trackers among community-dwelling older adults: Mixed method study. JMIR Mhealth Uhealth.

[56] Kruse CS, Mileski M, Moreno J (2017). Mobile health solutions for the aging population: A systematic narrative analysis. J Telemed Telecare.

[57] BinDhim NF, McGeechan K, Trevena L (2014). Who uses smoking cessation apps? A feasibility study across three countries via smartphones. JMIR Mhealth Uhealth.

